# Synthesis and Characterization of Hybrid-Magnetic Nanoparticles and Their Application for Removal of Arsenic from Groundwater

**DOI:** 10.1155/2013/387458

**Published:** 2013-09-30

**Authors:** Marta A. Bavio, Adriana G. Lista

**Affiliations:** FIA Laboratory, Analytical Chemistry Section, INQUISUR (UNS-CONICET), Avenue Alem 1253, B8000CPB Bahía Blanca, Buenos Aires, Argentina

## Abstract

Multiwall carbon nanotubes (MWCNTs) were oxidized with different agents and a characterization study was carried out. Then, hybrid-magnetic nanoparticles (HMNPs) were synthesized as iron oxide supported on the selected multiwalled carbon nanotubes (MWCNTs-Fe_3_O_4_) obtained from MWCNTs oxidized with HNO_3_. The HMNPs characterization revealed the presence of iron oxide as magnetite onto the MWCNTs surfaces. These HMNPs were used for arsenic removal from groundwater. The adsorption process variables were optimized (concentration of NPs, contact time, and pH), and these systems could remove 39.93 mg As/g adsorbent. Therefore, these nanoparticles appear as a good alternative for removing arsenic from water samples.

## 1. Introduction 

Arsenic is a toxic element and its presence in drinking water has caused the spread of endemic regional chronic hydroarsenicism (HACRE), evolving into more serious diseases like cancers, especially skin, lung, and liver cancer and lymphoma. The routes of exposure to humans are basically throughout the air, drinking water, or food [1]. High arsenic concentration can be found in drinking well water in many countries such as New Zealand, Romania, and Argentina. It is well known that new water sources are necessary, and the search is focused mostly on underground. Unfortunately, deterioration of this kind of water sources is observed. As an example, it can be mentioned the areas of La Pampa (Argentina), where it can be found aquifers with high contents in salt and arsenic (total dissolved solids greater than 1,400 ppm and 600–1200 ppb, resp.) [2]. Since 2007, the World Health Organization recommends a maximum concentration level of 10 *μ*g total arsenic L^−1^ [3] for drinking water (in 2004 the maximum was 50 *μ*g L^−1^).

The arsenic concentration in groundwater is usually controlled by natural geochemical processes, where adsorption-desorption reactions of As species on mineral surfaces play a key role. Adsorption-desorption processes are also important to develop water purification technologies, with the aim of removing As from water [4–7]. Arsenate adsorption has been studied using a variety of adsorbents. Most of the studies were performed using Fe(III) (hydr)oxides, which show highly selective adsorption capacities [8–16]. There is also considerable information about arsenate adsorption on phyllosilicates due to their high surface area. However, arsenate adsorption on these materials is lower than on minerals containing iron [17–20]. 

Magnetic carbon nanotubes composites are hybrids of magnetite (Fe_3_O_4_) and/or maghemite (**γ**-Fe_2_O_3_) with single-walled (SWCNTs) or multiwalled carbon nanotubes (MWCNTs). These composites combine the unique optical, electrical, and mechanical properties of carbon nanotubes (CNTs) and the paramagnetic or ferromagnetic properties of hybrid-magnetic nanoparticles (HMNPs) at room temperature. Such advantages have enabled their use as tips for magnetic force microscopes, separators in wastewater treatment, biosensors, drug delivery systems, and biomanipulators [21–24]. Magnetic CNTs can be prepared in different ways. A method involving the synthesis *in situ* of HMNPs onto the surface of CNTs holds much promise for large-scale synthesis [25–28]. Tan et al. [29] prepared magnetic CNTs by dispersing nanotubes in iron pentacarbonyl for their vacuum thermolysis and oxidation. Another method for *in situ* preparation of MNP-CNT composites involves decomposing ferrocene at a high temperature (350–500°C) onto previously purified CNTs. Chemical precipitation has also been used for *in situ* decoration of CNTs; the nanotubes are first oxidized in order to avoid breaking the tubes, and the process can be performed at fairly low temperatures [30]. Huiqun et al. [31] used chemical precipitation to decorate carbon nanotubes with iron oxide particles; the resulting hybrid particles spanned a broad range of sizes [32]. 

The aims of this work were to obtain magnetic nanoparticles and to carry out their characterization in order to use them as arsenic sorbent. Therefore, to synthesize the magnetic nanoparticles, a reaction onto the CNT surfaces was carried out by using a simple one step at high temperature. The precursor (iron chloride) and MWCNTs were homogenized in ethyleneglycol [33]. Although a high temperature was needed for efficient decoration, the reaction was relatively selective and sensitive. The product was characterized by using different techniques, and the obtained HMNPs were used in the targeted analysis for removing arsenic from contaminated groundwater. 

## 2. Experimental 

### 2.1. Apparatus

An Agilent HP 8453A UV-VIS spectrophotometer, with linear photodiode arrangement and a glass cell (1 cm of optic path), was used to quantify arsenic. Also a Perkin Elmer Optima 7000 atomic emission spectrophotometer with inductively coupled plasma was used.

The MWCNTs and HMNPs characterization was carried out using a Nicolet FTIR spectrophotometer, model Nexus 470; a scanning electronic microscope (SEM) LEO EVO 40 XVP model. Malvern Zetasizer Nano ZS90 was used for the zeta potential measurements. Powder X-ray diffraction was measured with a Phillips PW1710 diffractometer between 10° and 70° 2*θ* using CuK*α* radiation.

An ultrasonic bath Cole Palmer, a Gilson Minipuls 3 peristaltic, a pH-meter pH/ISE Meter Orion, and an analytical balance Ohaus AS120E were also used. A neodymium rare earth metal magnet disk of approximately 117,7 N in strength was applied for HMNPs separation.

### 2.2. Reagents and Solutions

All solutions were prepared using analytical reagent-grade and ultrapure water (18.0 m Ω cm^−1^, Milli-Q).

A 100 *μ*mol L^−1^ stock solution of arsenate was prepared by dissolving 0.0156 g of Na_2_HAsO_4_·7H_2_O (Merck, Argentina) in water to make up to 50 mL. The standard solutions were daily prepared by diluting the stock solution with water. The color reagent for the arsenate spectrophotometric determination was prepared according to Tsang et al. [34]. A solution of ascorbic acid (0.57 mol L^−1^) was prepared by dissolving 1.08 g ascorbic acid (Cicarelli, Argentina) in water and making up to 10 mL. This solution must be prepared and kept at 4°C. In these conditions, the solution can be used for 7 days.

Buffer solution of pH 2 was prepared by mixing 0.16075 g of citric acid (Cicarelli, Argentina), 0.0895 g of sodium chloride (Cicarelli, Argentina), and 0.205 mL of 1 mol L^−1^ hydrochloric acid (Merck, Argentina).

MWCNTs (>95% purity) were purchased from Bayer, Spain: inner diameter of 13–16 nm and 1–10 *μ*m of length.

### 2.3. Purification and Functionalization of MWCNTs

A thermal purification step of the MWCNTs was carried out at 350°C in a flask for 30 min. Then, these nanotubes were treated with HNO_3_ 4 mol L^−1^ for 2 h at room temperature to remove residual catalyst metals. They were washed with ultrapure water and dried at 110°C for 24 h.

In a second step, they were functionalized with different oxidants as it is indicated below.

- Refluxing with concentrated HNO_3_ at 150°C for 2 h [35].
- Heating with 0.5 mol L^−1^ KMnO_4_ at 80°C for 3 h [36].
- Heating with a solution containing HNO_3_ and HSO_4_ in a ratio 1 : 3, at 85°C for 3 h [37].
- Suspension with a solution of 70% NaOCl, stirring for 20 min and then heating at 85°C for 3 h [38].

In all cases, the obtained MWCNTs were washed with ultrapure water and dried at 110°C for 24 h. Then, they were characterized by IR spectroscopy and zeta potential measurements. 

### 2.4. Hybrid-Magnetic Nanoparticles (HMNPs) Synthesis

The characterization of the oxidized MWCNTs indicated that those treated with HNO_3_ were the best for further modification with iron.

HMNPs were synthesized following a similar procedure described by Zhan et al. [39]. Briefly, an amount of 14 mg of FeCl_3_·6H_2_O and 1 mg of the selected MWCNTs was suspended in 0.75 mL of ethyleneglycol in a glass vial. This solvent was used to provide monodispersed Fe_3_O_4_ nanoparticles. Then, 0.036 g sodium acetate was added and dissolved in order to get the electrostatic stabilization and to prevent particles agglomeration. This solution was kept at room temperature for 30 min. Then, the glass vial was placed in an airtight steel container and heated in an oven at 200°C for 16 h. After cooling to room temperature, the synthetic product was washed with 1 mL of water, and nanoparticles were recovered by applying a magnetic field with a magnetic disk placed on the outer wall of the glass vial. This cleanup procedure was repeated 5 times. The nanoparticles thus obtained can be stored in a dessicator until being used.

The general equations for the reaction are

(1) Fe2++2Fe3++8OH−+MWCNTs →MWCNTsFe3O4+4H2O

### 2.5. As(V) Adsorption Procedure

The obtained HMNPs were used to remove the As(V) from water samples. For this purpose, a suitable amount of the nanoparticles was placed in a glass tube with a solution containing a determined concentration of As(V). It was necessary to work at pH 2 to improve the As(V) adsorption on the HMNPs. The mixture was sonicated during 3 hours and then the hybrid-magnetic nanoparticles were isolated by using a magnetic field. In this way, the removal of the magnetic nanoparticles from solution was more selective and efficient (and often much faster) than centrifugation or filtration [40].

The concentration of As(V) in the liquid phase was spectrophotometrically determined following the modified method based on the use of molybdenum blue [34].

## 3. Results and Discussion

### 3.1. Characterization of Oxidized MWCNTs

#### 3.1.1. IR Spectroscopy

**Figure 1:**
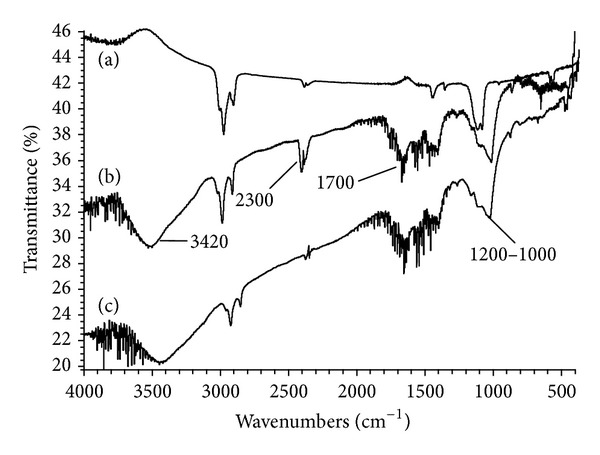
IR Spectra of purified and oxidized MWCNTs with (b) HNO_3_ and (c) NaOCl.

Figure 1 shows the IR spectra of purified and oxidized MWCNTs with the different oxidants. The spectrum of purified MWCNTs only exhibits peaks of –CH_2_ group because of the aromatic structure of nanotubes. On the other hand, the MWCNTs treated with NaOCl and HNO_3_ present four peaks at ~3420, 2300, 1700, and 1200–1000 cm^−1^. These peaks correspond to the tension bands of the hydroxyl (–OH), carboxyl (–COO–), carbonyl (–C=O), and C–O bond, respectively. This fact confirms the chemical modification on the surface of nanotubes with acid group. The spectra of the nanotubes oxidized with KMnO_4_ and 3N + 1S did not present differences from the purified nanotubes.

#### 3.1.2. Zeta Potential

**Figure 2:**
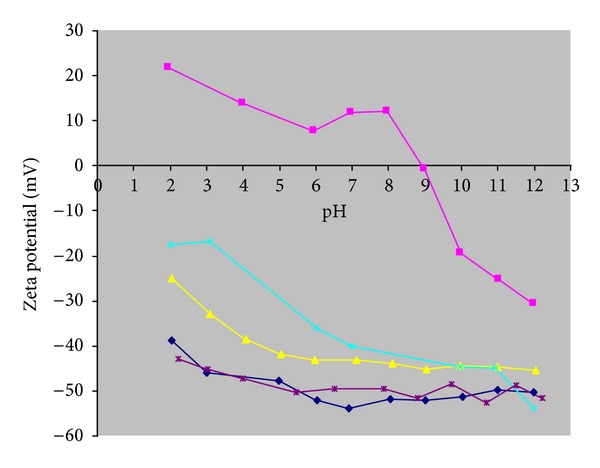
Zeta potentials of purified (■) and oxidized MWCNTs with HNO_3_ (♦); 3N + 1S (X); KMnO_4_ (▲); NaOCl (∗), under various pH.

The zeta potential is the electrical potential that exists in the liquid/solid interface. The development of electrical charge on the particle surface can affect the distribution of ions in a neighboring interfacial region, causing an increase in concentration of counterions near the surface.  Figure 2 shows the zeta potentials of purified and functionalized MWCNTs with different oxidants versus pH. As the pH increased, the surface charge of MWCNTs becomes more negative because there are more acid groups that lose protons. Where the zeta potential curve versus pH crosses the *x*-axis, isoelectric point (IEP) is obtained. When the pH is lower than IEP, the acidic solution donates more protons than the surface. Above IEP, the reverse situation takes place.

For Zeta potential measurements, 25 mg of MWCNTs is dispersed in 50 mL of ultrapure water and sonicated during 20 min. Before each measurement, the pH is adjusted between 2 and 12 with 0.1 M HCl or NaOH solutions. The ionic strength in this study is kept constant using a 0.02 mol L^−1^ NaCl solution. As can be seen in Figure 2, the zeta potential of purified MWCNTs is positive at pHs below the IEP and negative above this point. On the other hand, all the oxidized MWCNTs have negative zeta potential at all the tested pHs, and those modified with HNO_3_ and NaOCl show the lowest values. This indicates that more acidic groups were added to the surface, and then further analyses were carried out using those that were oxidized with HNO_3_.

#### 3.1.3. SEM Images of Purified and Oxidized MWCNTs with HNO_3_

**Figure 3:**
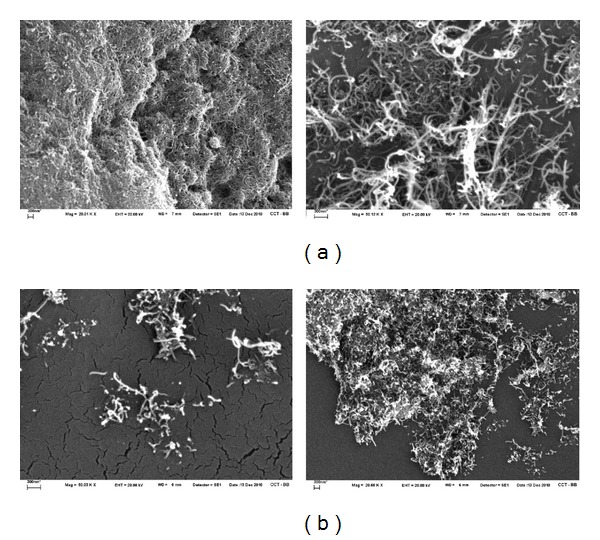
SEM images of MWCNTs (a) purified at 20000x and 50000x and (b) oxidized with HNO_3_ at 20000x and 50000x. The scale in all figures is 300 nm.

Figures 3(a) and 3(b) show the SEM images at different scales of the purified and oxidized MWCNTs, respectively. The first one shows large and curve nanotubes that are much agglomerated because of the Van der Walls forces. In the second one, it was observed that the agglomeration is lower, and the nanotubes are shorter because of the drastic acid treatment. A break in the defects of the tube is produced, and the ends of nanotubes are opened because there is more tension in the cycles of five atoms of carbon located there. 

### 3.2. Characterization of HMNPs

#### 3.2.1. IR Spectroscopy, X-Ray Diffraction, and Zeta Potential

**Figure 4:**
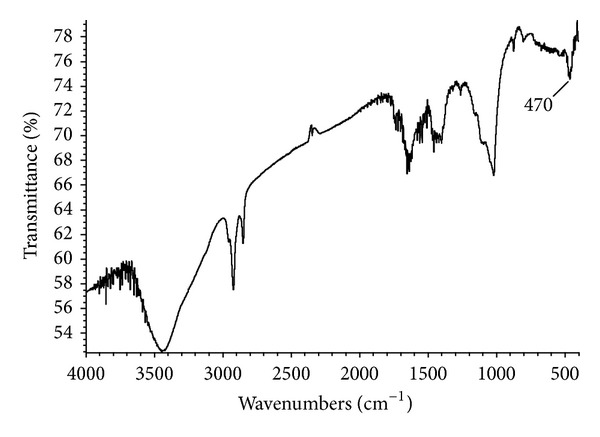
IR Spectra of HMNPs: oxidized MWCNTs decorated with Fe_3_O_4_.

Figure 4 shows IR spectra of the HMNPs. Peaks corresponding to the functional groups added after the chemical treatment are observed, and one new peak is identified at 470 cm^−1^ due to Fe–O bond resulting from the modification with iron [41].

The zeta potential of the hybrid nanoparticles was positive at pH lower than 3.0 (i.e., the IEP) and negative at pH over this value. This change indicates the adsorption of the iron oxide on the MWCNTs surface. So, the best pH values for the adsorption of arsenate are below pH 3, where the surface of the HMNPs is positive and, therefore, anions are more attracted.

It should be noted that the natural magnetite IEPs reported in the literature are between 5 and 6.8 [42], different to those obtained for the synthesized HMNPs. This fact is due to the magnitude of the negative zeta potential of the precursor MWCNTs. 

**Figure 5:**
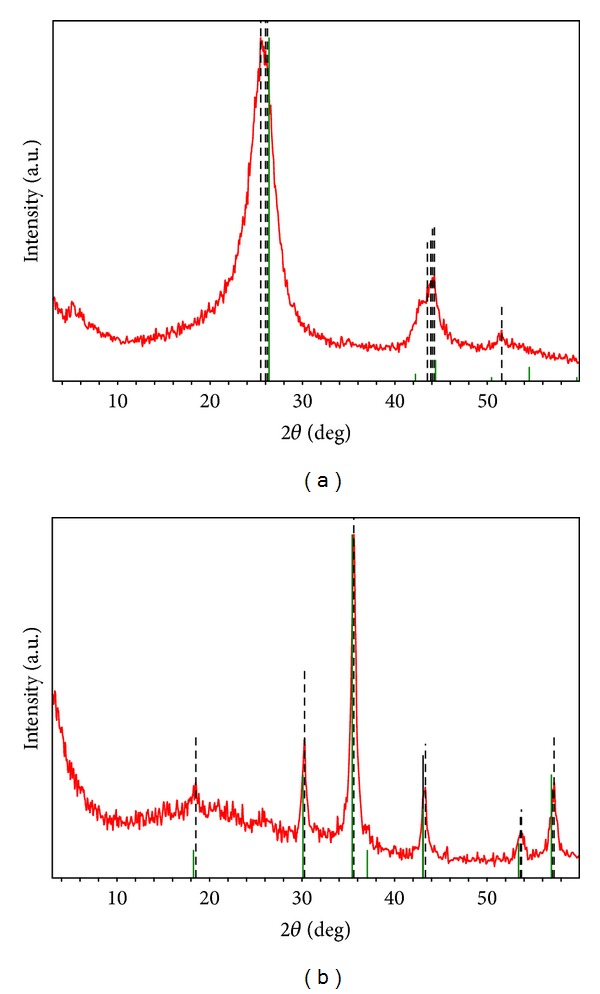
XRD patterns of (a) oxidized MWCNTs with HNO_3_ and (b) HMNPs.

The XRD patterns of MWCNTs oxidized with HNO_3_ and iron-decorated MWCNTs are given in Figures 5(a) and 5(b). In Figure 5(a), the peak at 2*θ* = 26.2° indicates the typical signal of carbon nanotubes or graphite structures. This peak is associated with the (002) diffraction of the hexagonal graphite structure in the carbon materials.  Figure 5(b) shows that a new hybrid magnetic structure was synthesized and had the chemicals composition of Fe_3_O_4_ (magnetite). New peaks at 18.5°, 30.3°, 35.6°, 43.1°, 53.6°, and 57.0° were observed. The positions match well with (111), (220), (311), (400), (422), and (511) planes of the standards XRD data of the cubical spinal crystal structure of magnetite. The principal peak of graphene structure at 26.2° did not appear probably because of the high ratio of magnetite composite with respect to carbon nanotubes. One peak that can be assigned to the graphene structure at 43.3° is distinguished, corresponding to the (101) plane.

#### 3.2.2. SEM Images of HMNPs

**Figure 6:**
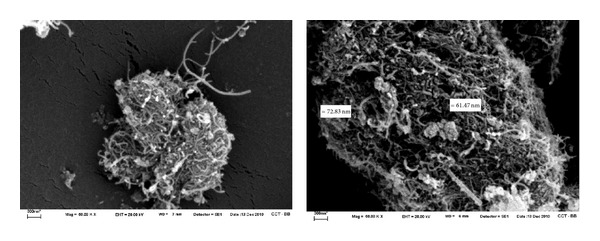
SEM images of HMNPs at 50000x. The scale in all figures is 300 nm.

Figure 6, show the SEM images at different scales of the iron-decoratived MWCNTs. They show short and straight nanotubes with little clusters of iron oxide with a diameter of about 60 nm. 

All of these analyses indicate that iron, as magnetite, is adsorbed on the oxidized MWCNTs. So, the content of iron in HMNPs was determined.

#### 3.2.3. Content of Iron in HMNPs

The iron content of the studied solids was determined by extraction with HCl [43]. 50 mg of solid was treated with 10 mL of concentrate HCl at 60–80°C during 1 hour. Then, the iron content in the separated supernatant was determined by using the spectrophotometric tiocianate method at 475 nm [44]. The obtained result was 234.5 mg Fe^+3^/g of HMNPs, which means that 23.4% of the nanoparticles corresponds to iron.

### 3.3. As(V) Adsorption Procedure Optimization

For this purpose, pH, contact time, and amount of HMNPs were studied. It is well known that it is necessary to achieve the equilibrium of the adsorption process, and, for this, the contact time between the HMNPs and the analyte is crucial. 

The pH is other critical variable because the HMNPs surficial charge and the behavior of the As species depend on it. Finally, the concentration of HMNPs dispersed in the solution must be adequate in order to have the maximum surface available for the interaction with the arsenate ions. 

The optimization of the mentioned variables was carried out by the univariant way and using the calibration curve obtained with the modified molybdenum blue method. The linear range for the straight line (*y* = 0.0003 *μ*g L^−1^–0.0018; *R* ^2^ = 0.998) was between 40 and 600 *μ*g As(V) L^−1^. A 600 *μ*g L^−1^ arsenic solution was used as initial concentration for the study of each variable, considering that the absorbance measurements were carried out on the supernatant solution after the adsorption procedure. The optimum value for each variable was selected considering the greatest difference between the As(V) concentration before and after the adsorption procedure.

The studied ranges for the variables and the optimal values for each one appear in Table 1.

Working under these conditions, a maximum of 53 mg As(V)/g of HMNPs can be removed from contaminated solutions. This value is lower than the result reached with akaganeita (*β*-FeOOH) (120 mg As/g) [45]. However, it is closer to the ones obtained with ferrihydrite (5Fe_2_O_3_·9H_2_O) and maghemite (i.e., 52 and 50 mg As/g, resp.) [46, 47] and higher than the results obtained when zeolite dopped with Fe is used (35 mg As/g) [48].

### 3.4. Arsenic Determination in Real Samples

Two groundwater samples from different zones of the Bahía Blanca city were analysed. The determination of As in real samples was done by ICP. The analyses were carried out on samples with and without adsorption procedure using HMNPs, and the results are shown in Table 2. 

As can be seen, the amount of adsorbed arsenic is different in both samples. When the concentration is higher, the percentage of adsorbed arsenic is higher too. This fact is consistent with previous publications, which indicate that the adsorption of As on natural magnetic nanomaterials decreases when the initial concentration of As in the sample is lower [49].

It is clear that the adsorption process occurs in real samples with both species of As(V) and As(III). It should be remembered that the As(V) binds more strongly to Fe or Mn oxides, compared with the species of As(III). Also, the adsorptive affinity for As(V) is higher at acid pH, and for As(III) alkaline conditions are more favourable [50].

## 4. Conclusions

The functionalization of multiwall carbon nanotubes was verified with different characterization techniques. Those treated with HNO_3_ were selected as substrate for the preparation of HMNPs. The modification of this MWCNTs with Fe (III) salt resulted in an hybrid nanoparticle (combination of MWCNTs and iron oxide nano particles) with a high capability for arsenic adsorption. On the other hand, the magnetic behavior of these nanoparticles makes possible their separation from the sample solution after the treatment.

Even though the synthesized HMNPs adsorb less than some natural adsorbents as akaganeite, the obtained results make it possible to conclude that this nanotechnology is an original and potential solution for arsenic removal from superficial and groundwater samples.

## Figures and Tables

**Table 1:** Adsorption process: optimization of variables.

	Studied range	Selected value	mg As(V)/g HMNPs
Contact time (h)	1–6	3	32.70
Amount of adsorbent (mg)	1–7	3.5	37.97
pH	2–7	2	39.93

**Table 2:** Analysis of real groundwater samples.

Groundwater samples	Palihue	Patagonia
Total As concentration founded (*µ*g·L^−1^)		
Untreated sample	37 ± 0.5	353 ± 1.9
Treated sample*	25.5 ± 0.7	165.5 ± 2.1
Total As adsorbed (mg As/g HMNPs)	31.00	53.11

*Average of three replicates.
